# Magnetocaloric effect and negative thermal expansion in hexagonal Fe doped MnNiGe compounds with a magnetoelastic AFM-FM-like transition

**DOI:** 10.1038/srep41675

**Published:** 2017-01-30

**Authors:** Kun Xu, Zhe Li, Enke Liu, Haichun Zhou, Yuanlei Zhang, Chao Jing

**Affiliations:** 1 Research Center for Magnetic Materials and Devices, Key Laboratory for Advanced Functional and Low Dimensional Materials of Yunnan Higher Education Institute, Qujing Normal University, Qujing, 655011, China; 2 State Key Laboratory for Magnetism, Beijing National Laboratory for Condensed Matter Physics, Institute of Physics, Chinese Academy of Sciences, Beijing, 100190, China; 3 Department of Physics, Yunnan Normal University, Kunming, 650500, China; 4 Department of Physics, Shanghai University, Shanghai, 200444, China

## Abstract

We report a detailed study of two successive first-order transitions, including a martensitic transition (MT) and an antiferromagnetic (AFM)-ferromagnetic (FM)-like transition, in Mn_1-*x*_Fe_*x*_NiGe (*x* = 0, 0.06, 0.11) alloys by X-ray diffraction, differential scanning calorimetry, magnetization and linear thermal expansion measurements. Such an AFM-FM-like transition occurring in the martensitic state has seldom been observed in the *M(T*) curves. The results of Arrott plot and linear relationship of the critical temperature with *M*^2^ provide explicit evidence of its first-order magnetoelastic nature. On the other hand, their performances as magnetocaloric and negative thermal expansion materials were characterized. The isothermal entropy change for a field change of 30 kOe reaches an impressive value of −25.8 J/kg K at 203 K for *x* = 0.11 compared to the other two samples. It demonstrates that the magneto-responsive ability has been significantly promoted since an appropriate amount of Fe doping can break the local Ni-6Mn AFM configuration. Moreover, the Fe-doped samples reveal both the giant negative thermal expansion and near-zero thermal expansion for different temperature ranges. For instance, the average thermal expansion coefficient *ā* of *x* = 0.06 reaches −60.7 × 10^−6^/K over *T* = 231–338 K and 0.6 × 10^−6^/K over *T* = 175–231 K during cooling.

The family of hexagonal *MM*’*X (M, M*’ = transition metals, *X* = carbon or boron group elements) compounds has been extensively investigated over the past few decades^1^,^2^,^3^,^4^. Among these materials, the stoichiometric MnNiGe alloy undergoes separate magnetic and crystallographic transitions during cooling and is absent of a first-order magnetostructural phase transition (FOMST). In recent years, numerous successful attempts have been made to tune these two separate transformations simultaneously to coincide through the chemical modification^3^,^4^,^5^,^6^,^7^,^8^,^9^, physical pressure^10^,^11^, or alternation of sample form (from bulk to ribbon)^12^,^13^, and the introduction of atom vacancies^14^. During the cooling process, such a coincidence can arouse a FOMST from a paramagnetic (PM) Ni_2_In-type hexagonal austenitic to an antiferromagnetic (AFM) or a ferromagnetic (FM) TiNiSi-type orthorhombic martensitic phase. This behavior has made these alloys gain considerable scientific and technological interest in the areas of the magnetocaloric effect (MCE)^6^,^7^,^8^,^9^,^10^,^11^,^15^ and negative thermal expansion (NTE)^16^.

As previously pointed out by Szytula *et al*.^2^ and Landrum *et al*.^17^, the TiNiSi-type martensitic structure of *MM*’*X* is particularly complex (up to 495 manifestations with six-rings in chair or boat forms). Because of the different spatial configurations, the magnetic arrangements also reveal polymorphism, including simple spiral AFM, cycloidal spiral AFM, FM spiral, non-collinear FM and collinear FM, as identified by neutron diffraction measurements^18^,^19^. This can be attributed to the fact that their magnetic exchange interaction strongly depends on the spatial separation of Mn atoms. Another appealing magnetic property is the existence of an abnormal transition from a cycloidal spiral AFM to a simple spiral AFM in the martensitic phase of stoichiometric MnNiGe involving a reorientation of spiral axis^18^. With Co substituting on the Ni site, this transition gradually converts to a “spiral AFM – canted FM” one^18^,^20^ due to the enhanced FM interaction. This transition was usually regarded as a simple second-order magnetic transition. Moreover, the isothermal magnetization curve in the MnNi_1-*x*_Fe_*x*_Ge ribbon/bulk exhibits a magnetically reversible AFM-FM transition at low fields without any hysteresis^12^,^15^, which implies the possibility of its second-order nature. On the contrary, Trung *et al*. suggested that such an AFM-FM transition in polycrystalline Mn_0.75_Cr_0.25_CoGe alloys should be classified as a first-order magnetoelastic one, as evidenced by the prominent thermal hysteresis in the *M(T*) curves, and a sudden change into Invar-like behavior below a critical temperature^21^. In the aforementioned systems, Co or Fe would induce FM ordering in the intrinsically spiral AFM MnNiGe^6^, while the Cr substitution in the originally collinear FM MnCoGe would induce AFM ordering^21^. All of them will result in a competition between FM and AFM consequently.

The aim of this study is to let the FOMST and the AFM-FM-like transition to occur successively in one system. To realize this goal, firstly, an appropriate amount of Fe-doping could reduce the temperature of martensitic transition (MT) within the “Curie temperature window”^6^ and form a FOMST. Secondly, not too much Fe should be introduced since the AFM-FM-like transition will vanish if FM interaction dominates. In this line of reasoning, we have chosen Mn_1-*x*_Fe_*x*_NiGe (*x* = 0, 0.06, 0.11) as our object of study by referring the phase diagram of Fe-doped MnNiGe^6^. The origin of the transition behaviors in these samples was addressed by means of X-ray diffraction (XRD), differential scanning calorimetry (DSC), magnetization and linear expansion measurements. On this basis, the MCE, thermal expansion and magneto-strain performances upon MT were also evaluated in these compounds.

## Results

### MT characterized by X-ray diffraction and calorimetry

#### XRD characterization

**Figure 1:**
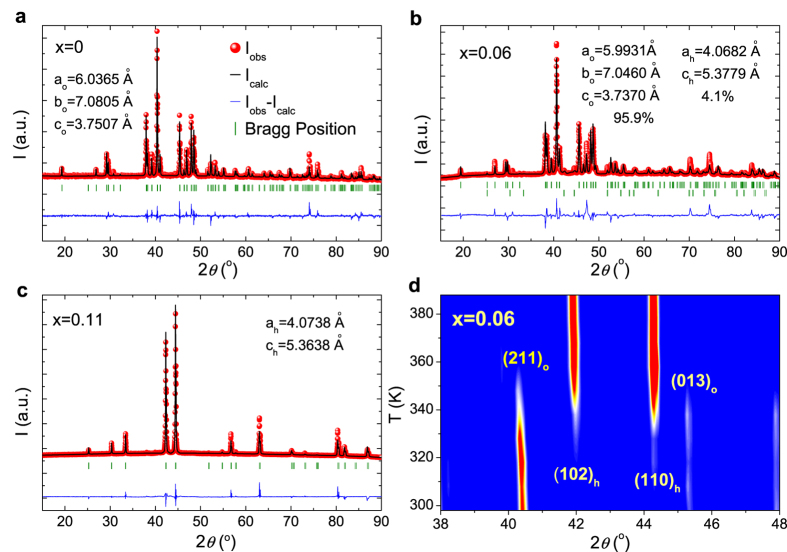
Room-temperature XRD patterns for the samples with (**a)** *x* = 0, (**b**) *x* = 0.06 and (**c**) *x* = 0.11 following the Rietveld fitting. (**d**) The contour plot of temperature dependent of the XRD patterns during a cooling cycle for the sample with *x* = 0.06.

Figure 1a–c show the Rietveld refined room temperature XRD patterns for the Mn_1-*x*_Fe_*x*_NiGe samples with *x* = 0, 0.06 and 0.11. At room temperature, the reflections of the samples with *x* = 0 and *x* = 0.11 can be satisfactorily indexed as a TiNiSi-type orthorhombic single phase and a Ni_2_In-type hexagonal single phase, respectively. The sample with *x* = 0.06 crystallizes predominately in the orthorhombic structure accompanied by 4.1% residual hexagonal phase. The refined result shows that the nearest Mn-Mn distance in the orthorhombic phase reduces from 3.1376 Å for *x* = 0 to 3.1319 Å for *x* = 0.06, and in the hexagonal phase it also decreases from 2.6890 Å for *x* = 0.06 to 2.6819 Å for *x* = 0.11. These findings indicate that the positive “chemical pressure”^22^,^23^ induced by replacing larger Mn atoms by smaller Fe can stabilize the high-temperature hexagonal phase due to the reduction of the Mn-Mn distance^6^,^24^,^25^. In addition, Mn_1-*x*_Fe_*x*_NiGe with *x* = 0.06 was also used as a representative sample to characterize the structural evolution with temperature in detail. Figure 1d presents its 2-dimensional contour image of the XRD patterns collected during a cooling cycle from 388 K to 298 K. It can be clearly seen that this sample undergoes a structural transition from the hexagonal to the orthorhombic phase upon cooling. The lattice parameters show a sharp discontinuity in the vicinity of 338 K, which leads to a remarkable increase of 2.81% in the unit-cell volume during the forward MT, based on the relation *a*_*orth*_ = *c*_*hex*_, *b*_*orth*_ = *a*_*hex*_, 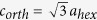 and *V*_*orth*_ = 2*V*_*hex*_^19^. Such a large value of volume change is in satisfactory agreement with that reported for the Mn_0.84_Fe_0.16_NiGe alloy^6^.

#### DSC characterization

**Figure 2:**
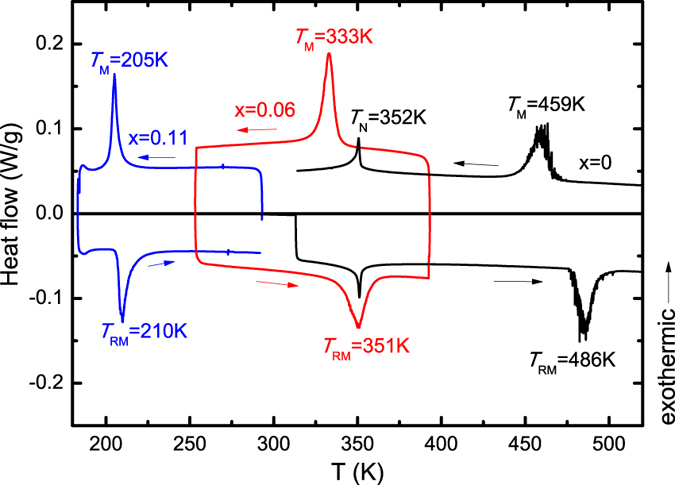
The DSC scans for the samples with *x* = 0, 0.06 and 0.11 with a measuring rate of 3 K/min.

The occurrence of MT was examined by the DSC measurements for Mn_1-*x*_Fe_*x*_NiGe (*x* = 0, 0.06 and 0.11), as shown in Fig. 2. In agreement with other reports^3^,^4^,^6^, the undoped sample undergoes two separate transitions including a PM-PM forward MT at *T*_M_ = 459 K (reverse MT at *T*_RM_ = 486 K) and a PM-AFM magnetic transition at the Néel temperature *T*_N_ = 352 K. For the Fe-doped samples, only the forward and reverse MTs are evidenced from the well-defined exothermic/endothermic peaks in the heat flow curves, arising from the latent heat of the transitions. The characteristic temperatures are determined to be *T*_M_ = 333 K and *T*_RM_ = 351 K with a hysteresis of 18 K for *x* = 0.06, as well as *T*_M_ = 205 K and *T*_RM_ = 210 K with a hysteresis of 5 K for *x* = 0.11, respectively. Both *T*_M_ and thermal hysteresis are significantly reduced with increasing Fe doping. From the baseline corrected calorimetric data, the transition entropy change (Δ*S*_*tr*_) can be estimated using the following relationship:


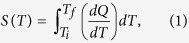


where *T*_*i*_ and *T*_*f*_ are the initial and final temperature limits of integration, respectively. Using Equation (1), the Δ*S*_*tr*_ values contributed from the forward MT are found to be ~−29.1 J/kg K, ~−51.3 J/kg K and ~−42.5 J/kg K, respectively. Δ*S*_*tr*_ corresponds to the complete transition, and is the maximum achievable value of entropy change induced by temperature, magnetic fields or pressure^26^. This implies that an impressive isothermal entropy change (Δ*S*_*T*_) can be encountered by applying a sufficiently strong magnetic field variation to this system.

### Multiple transforming behaviors characterized by magnetization measurements

#### M(T) curves

**Figure 3:**
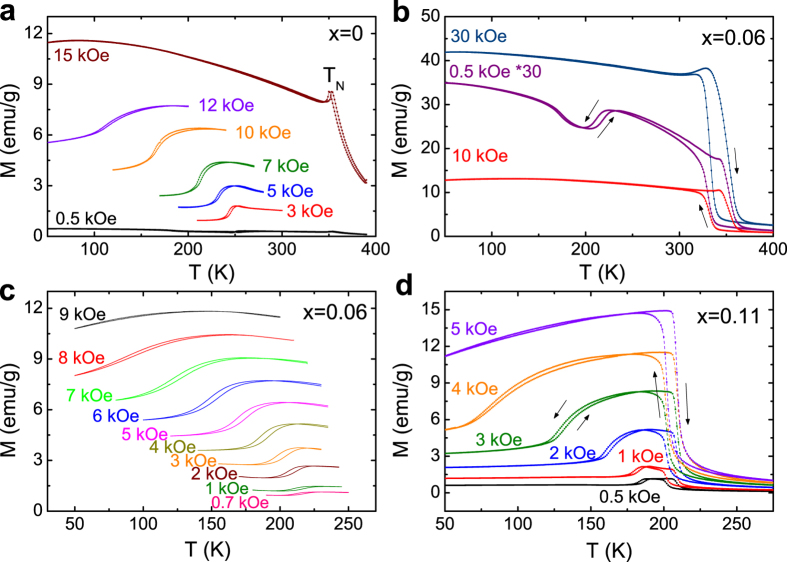
*M(T*) curves under typical magnetic fields for the sample with (**a**) *x* = 0; (**b**) and (**c**) *x* = 0.06; (**d**) *x* = 0.11. In (**b**), the magnetization in the field of 0.5 kOe for *x* = 0.06 is enlarged by a factor of 30.

To examine the phase transforming behaviors further, the temperature-dependent magnetization data (*M(T*)) for Mn_1-*x*_Fe_*x*_NiGe (*x* = 0, 0.06, 0.11) are collected. Figure 3a shows the *M(T*) curves measured under different magnetic fields for the undoped sample. Due to the absence of FOMST, only a sharp peak related to the AFM-PM transition is observed at *T*_N_ = 352 K in *H* = 15 kOe. While for the Fe-doped sample with *x* = 0.06, Fig. 3b shows the *M(T*) curves measured in the presence of *H* = 0.5 kOe, 10 kOe and 30 kOe. Sharp changes in magnetization occur around ~332 K for cooling and ~352 K for heating, associated with the forward and reverse MTs, respectively. These temperatures are consistent with those determined by calorimetric measurements (see Fig. 2). The considerable thermal hysteresis is a direct manifestation of the first-order nature of this transition. Moreover, the difference in magnetization between two phases is over 35 emu/g in the magnetic field of 30 kOe. This means that such a small amount of Fe doping has broken the perfect 6Mn-Ni antiferromagnetic spin arrangement, and induced FM coupling against the AFM interaction in the originally AFM-coupled martensitic matrix^6^. Strikingly, besides MT, another AFM-FM-like transition can also be distinguished unambiguously in the martensitic state in the field of 0.5 kOe. The critical temperature (*T*_*cr*_) determined from the inflection point is 212 K for cooling (218.5 K for heating) with a hysteresis of ~6.5 K. Such an abnormal transition may be a consequence of the competition between Fe-doping-induced FM and the intrinsic AFM exchange interactions. Due to the crystallographic polymorphism of the martensitic phase, the atom position may also vary concurrently with the magnetic transition^27^, showing a first-order magnetoelastic coupling characteristic. In order to explore this exotic behavior further, an overlay of *M(T*) curves under different applied fields in the range of 0.7−9 kOe was also measured for the sample with *x* = 0.06, as illustrated in Fig. 3c. The occurrence of this transition is limited to weak magnetic fields and is gradually suppressed with increasing field. This reveals that the re-entrant AFM-like structure at low temperatures is unstable, and an applied field can easily tilt the balance of the AFM-FM competition. This leads the *T*_*cr*_ to shift towards the lower temperatures significantly with the increase of magnetic field (see in Fig. 3c). In addition, several particular features can be also distinguished: (i) The transition spreads over a wider temperature range with increasing field; (ii) Both *T*_*cr*_ and the transition width reveal a stronger correlation with the external fields, while keeping the width of thermal hysteresis nearly unchanged with increasing field; and (iii) Such an AFM-FM-like transition in the *M(T*) curve almost disappears at *H* = 9 kOe for a lower-temperature-limit of 50 K. As a matter of fact, this transition is supposed to survive below 50 K under a field slightly above 9 kOe. Similar behaviors can be observed for the samples with *x* = 0 in Fig. 3a and *x* = 0.11 in Fig. 3d. For the sample with *x* = 0, this AFM-FM-like transition with a thermal hysteresis (~2.8 K) has been proven to be a cycloidal spiral AFM to simple spiral AFM transition by neutron diffraction measurement^18^. It occurs at a higher temperature (~250 K in *H* = 0.5 kOe) and disappears at a greater field (>12 kOe) compared to the alloy with *x* = 0.06. In contrast, for the alloy with *x* = 0.11, the AFM-FM-like transition with a thermal hysteresis (~3.4 K) occurs at a lower temperature (~184 K in *H* = 0.5 kOe) and vanishes at a much smaller magnetic field (5 kOe). The decrease of *T*_*cr*_ and vanishing field for the AFM-FM-like transition with more Fe can be understood by the fact that the FM exchange interaction is strengthened in the martensitic state. Notably, with the enhancement of the FM interaction, we speculate that the AFM-FM-like transition for the Fe-doped samples would be from the spiral AFM to the canted FM, as in the Co-doped MnNiGe^18^,^20^.

#### M(H) curves

**Figure 4:**
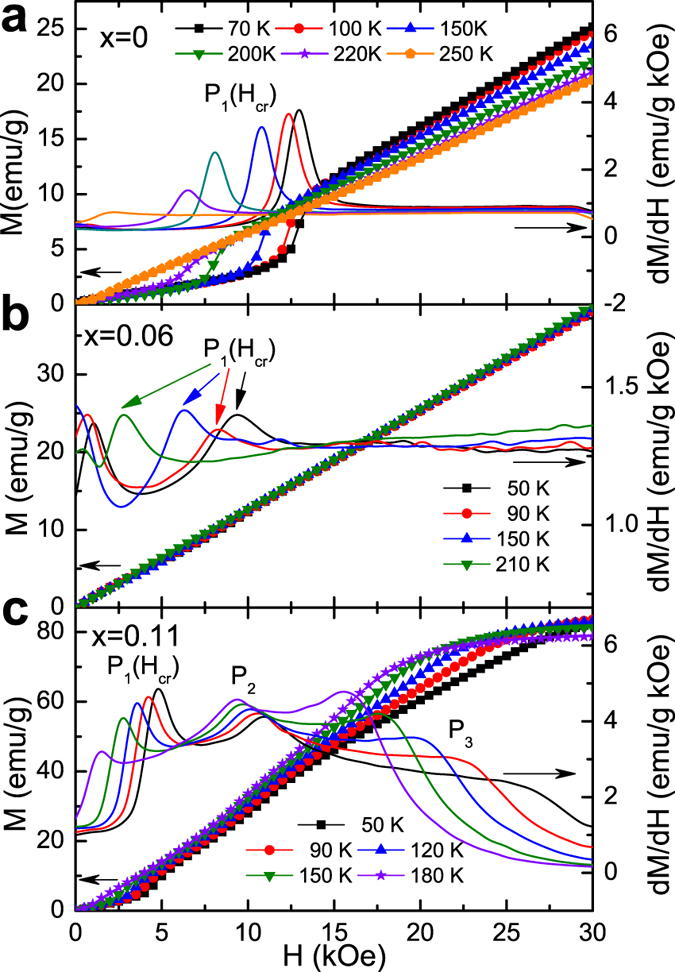
Isothermal magnetization (left-Y axis) and the corresponding d*M*/d*H* (right-Y axis) plots at selected temperatures for (**a**) *x* = 0, (**b**) *x* = 0.06 and (**c**) *x* = 0.11.

**Figure 5:**
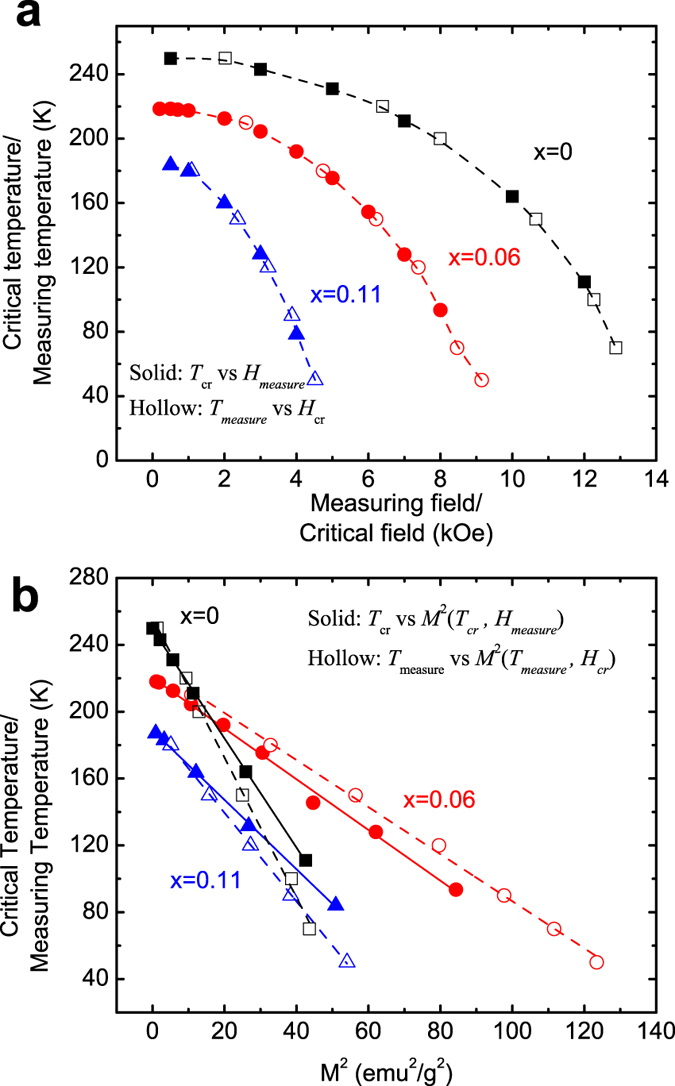
(**a**) The *T*_cr_ versus measuring field, and measuring temperature versus *H*_cr_ plots corresponding to the AFM-FM-like conversion (**b**) *T*_cr_ versus *M*^2^ plots for the samples with *x* = 0, 0.06 and 0.11.

In order to gain greater insight into the field dependence of this AFM-FM-like transformation, the isothermal magnetic measurements (*M(H*)) were implemented, as mentioned in most previous investigations^6^,^12^,^15^. Figure 4a–c give the measured *M(H*) and corresponding derivative d*M*/d*H* curves at selected temperatures for the alloys with *x* = 0, 0.06 and 0.11, respectively. One can notice that, all the samples undergo a transition at a critical magnetic field (*H*_*cr*_) in the *M(H*) curves. The *H*_*cr*_ is defined as the magnetic field corresponding to the peak “*P*_1_” in the d*M*/d*H* curve, indicating the occurrence of an AFM-FM-like metamagnetic transition in the martensite. This transition was also claimed to be a spiral AFM to canted FM transition in some MnNiGe-based systems^28^,^29^,^30^. On the basis of the fact that both *T*_*cr*_ and *H*_*cr*_ are the critical parameters for the AFM-FM-like transition in these samples, we extract them accurately from the *M(T*) and *M(H*) curves and plot the (*H, T*) diagram in Fig. 5a. It can be clearly observed that the *T*_*cr*_(*H*) and the *H*_*cr*_(*T*) follow the same trend, manifesting that the transition at *T*_*cr*_ in the *M(T*) curve is exactly the “*P*_1_” transition at *H*_*cr*_ in the *M(H*) curve for all these three samples.

Different to the first two alloys, the *M(H*) curve of the sample with *x* = 0.11 with stronger FM interaction contains other two inflection points, labeled “*P*_2_” and “*P*_3_”, in Fig. 4c. This suggests a complex process of AFM-FM competition and conversion. The peak “*P*_2_” may be a fingerprint of a transition from the FM-like state (canted FM) to a collinear FM state, as referred to the phase diagram in another study^18^ by neutron diffraction measurements. With further increases in the applied field, the magnetization of the martensitic state seems to reach saturation at “*P*_3_”.

**Figure 6:**
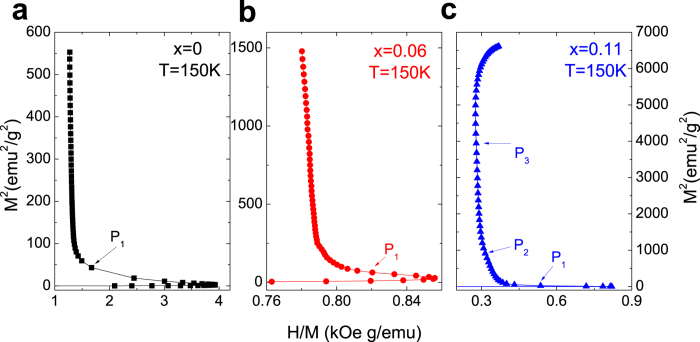
The standard Arrott plots at *T* = 150 K for the samples with (**a**) *x* = 0, (**b**) *x* = 0.06 and (**c**) *x* = 0.11.

Subsequently, the standard Arrott plot (*M*^2^-*H*/*M*) was carried out to clarify the nature of these complex metamagnetic transitions. The standard Arrott plots at 150 K for Mn_1-*x*_Fe_*x*_NiGe (*x* = 0, 0.06, 0.11) are depicted in the Fig. 6a–c. It is found that the “*P*_1_” and “*P*_2_” are located in the regimes with an obvious negative slope (marked by arrows), which confirms the first-order nature of both transitions. Consequently, we guess that the AFM-FM-like transition during the application of magnetic field for the sample with *x* = 0.11 is constituted by three successive stages, i.e., spiral AFM → canted FM → collinear FM by referring the phase diagram^18^.

#### Magnetoelastic interaction

The occurrence of the AFM-FM-like transition is assumed to be accompanied with the magnetoelastic interaction, suggested by a strong field dependence of *T*_*cr*_ (Fig. 5a). The presence of magnetoelastic interaction in another *MM*’*X* system, CoMnSi, has been proven by the neutron diffraction measurements^31^. According to the Landau-type model^32^, the magnetoelastic energy should be quasi-linear with *M*^2^. For the pre-martensitic transition in the stoichiometric Ni_2_MnGa alloy, the *T*_*cr*_ varies linearly with *M*^2^ due to magnetoelastic interaction^33^,^34^. Based on this consideration, we re-plotted the *T*_*cr*_ versus *M*^2^ curves using the *M(T*) data and the measuring temperature versus *M*^2^(*H*_*cr*_) curves from the isothermal *M(H*) data (Fig. 5b). From this figure, it is clear that all these curves present a linear relationship, which sufficiently supports the presence of a strong magnetoelastic coupling during the AFM-FM-like transition. However, the slopes from isofield measurements deviate slightly from those from isotherms. This is a consequence of the fact that the *M(T*) curves offer an extended range of temperatures and fields, whereby they are preferred for establishing the quasi-equilibrium (*T, H*) phase diagram^35^. Therefore, such a unique transition in the martensitic state can be attributed to the first-order magnetoelastic transition.

### Caloric and strain performance corresponding to the MT

#### Isothermal entropy change

Δ*S*_*T*_ associated with the FOMST was calculated by means of the integrated Maxwell relation using *M(T*) data to avoid the “spurious” spike^36^,^37^,


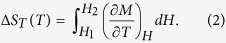


**Figure 7:**
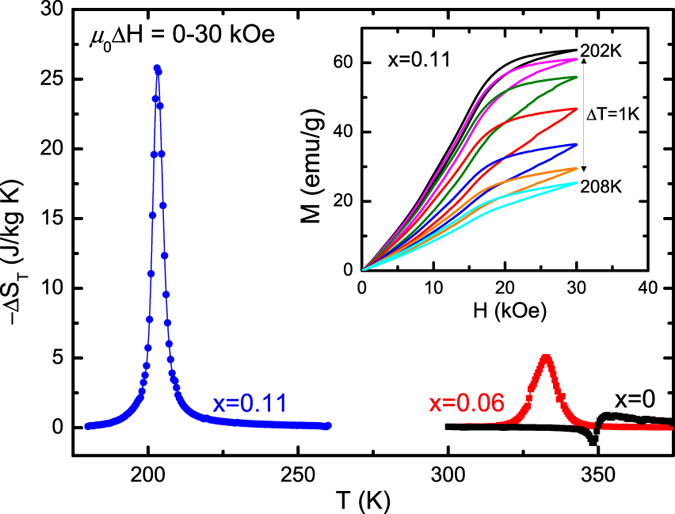
The Δ*S*_*T*_ determined by the integrated Maxwell relation for a field change *μ*_0_Δ*H*  = 0–30 kOe. Inset: The *M(H*) curves at several temperatures during the MT for the sample with *x* = 0.11.

Figure 7 describes the temperature dependence of Δ*S*_*T*_ during the forward MT for a magnetic field change of 30 kOe. For the undoped sample, the calculated Δ*S*_*T*_ is −0.9 J/kg K at 353 K related to the second-order PM-AFM transition. For the compound with *x* = 0.06, the FOMST occurs and the corresponding maximum Δ*S*_*T*_ is about –5.1 J/kg at 333 K. Further, an enhanced MCE is observed in the compound with *x* = 0.11, and its Δ*S*_*T*_ quickly climbs to ~–25.8 J/kg K at 203 K, which has also been verified by the Maxwell relation using isothermal *M(H*) data (inset of Fig. 7). This value is significantly higher than those in other *MM*’*X* compounds^12^,^15^,^37^,^38^,^39^ and is comparable to some giant magnetocaloric materials with FOMST^40^,^41^. The enhanced Δ*S*_*T*_ can be ascribed to the extremely narrow transition temperature width and the improved FM exchange interaction activated by the introduction of more Fe atom^6^.

#### Strain performance

**Figure 8:**
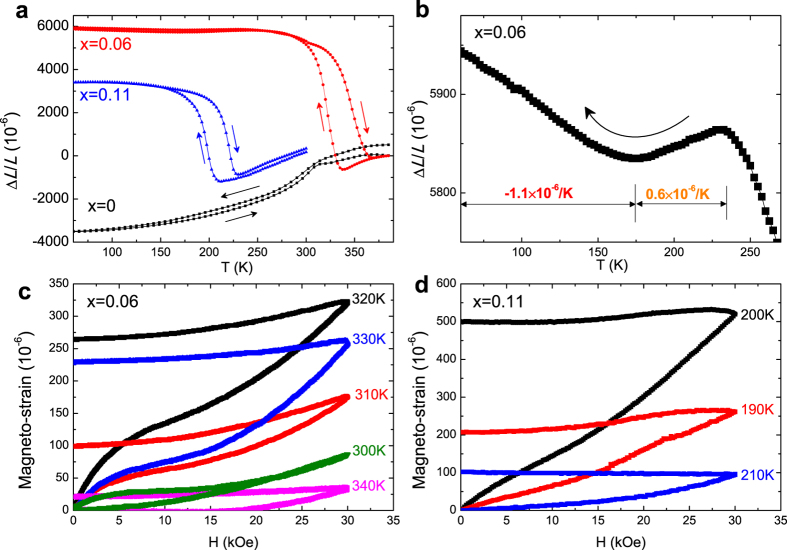
(**a**) The temperature dependences of Δ*L*/*L* for *x* = 0, *x* = 0.06 and *x* = 0.11 in the absence of magnetic fields. (**b**) The enlarged view of Δ*L*/*L* in the low-temperature region for *x* = 0.06. Magneto-strain curves for the samples with (**c**) *x* = 0.06 and (**d**) *x* = 0.11.

For Mn-Co-Ge-based^16^, La-Fe-Co-Si^42^, and Co-Mn-Si^29^,^43^ compounds, one of the most distinctive features is the pronounced NTE with a wide operation-temperature window, associated with a magneto-volume effect. From the high-temperature XRD measurements in Fig. 1d, lattice volume expands 2.81% upon cooling across the forward MT for the sample with *x* = 0.06. If this polycrystalline sample expands isotropically, the ideal linear NTE should reach a considerable value of ~9300 × 10^−6^. Hence, our samples in the powder form were bonded using 20 wt.% epoxy resin, which guarantees that the samples would not break into pieces to contaminate the vacuum chamber of Versalab. The linear thermal expansion Δ*L*/*L* curves for the bonded samples with *x* = 0, 0.06 and 0.11 were measured and plotted in Fig. 8a. As expected, no NTE behaviors are demonstrated for the undoped sample because the MT temperatures *T*_M_ = 459 K and *T*_RM_ = 486 K are out of the measured temperature range. For the sample with *x* = 0.06, it has a broad transition width that covers room temperature, and thus is favorable for the potential application as an NTE material. We can see from Fig. 8a that the variation of Δ*L*/*L* reaches ~6500 × 10^−6^ during the forward MT in the absence of magnetic fields, which is only ~70% of the predicted crystallographic value. This indicates that the abundant epoxy resin and the porosities that possibly formed during bonding processing seriously affect the strain performance of this sample. Compared to the colossal Δ*L*/*L* value of ~10000 × 10^−6^ for bonded MnCoGe-based compounds with ~4% variation in lattice volume during the MT, this value of ~6500 × 10^−6^ is relatively low. Nevertheless, such a value is comparable to other promising NTE materials (~6000 × 10^−6^ for Bi_0.95_La_0.05_NiO_3_^44^, ~3500 × 10^−6^ for La-Fe-Co-Si^42^ and ~4600 × 10^−6^ for anti-perovskite manganese nitride Mn_3_ZnN^45^). In addition, the average NTE coefficient *ā* of this sample reaches a giant value of −60.7 × 10^−6^/K with a wide operation-temperature window of 107 K (231–338 K), covering the room temperature. Furthermore, when the sample is cooled down to the martensitic state, the Δ*L*/*L* presents a near-Invar-like temperature independent behavior, as shown in the Fig. 8b. The absolute value of *ā* is below 1.1 × 10^−6^/K over a very broad temperature range (60–231 K). In particular, it shows a low expansion of 0.6 × 10^−6^/K over *T* = 175–231 K. Such excellent features ensure that it is a promising candidate for both NTE and zero-thermal-expansion^46^ materials. For the sample with *x* = 0.11, the Δ*L*/*L* reaches ~4500 × 10^−6^ upon cooling. The smaller Δ*L*/*L* in *x* = 0.11 is consistent with its smaller Δ*S*_*tr*_ compared to *x* = 0.06. Finally, the magneto-strain of the Fe-doped samples were evaluated, as shown in Fig. 8c and d. The maximum magneto-strains by applying a magnetic field of 3 kOe are only 320 × 10^−6^ and 530 × 10^−6^ for the samples with *x* = 0.06 and 0.11, respectively. This confirms the insensitivity of the MT to the magnetic field for Mn-Ni-Ge-based systems^47^.

## Discussion

Under low fields, a first-order magnetoelastic AFM-FM-like transition in the martensite state has been observed explicitly in the Mn_1-*x*_Fe_*x*_NiGe (*x* = 0, 0.06 and 0.11) system from both the *M(T*) and *M(H*) data. Despite numerous studies that have been reported on *MM*’*X* systems, there is still lack of the systematical investigation on the variation of this AFM-FM-like transition with temperature and magnetic field. And the direct evidence of its first-order magnetoelastic nature are rare. In both MnNi_1-*x*_Fe_*x*_Ge and Mn_1-*x*_Fe_*x*_NiGe systems with a visible AFM-FM-like transition in *M(T*) curves, either Fe-doping can change the original AFM-arranged MnNiGe matrix to a strong FM arrangement. Nonetheless, Fe-doping on the Ni site will result in a very narrow available temperature range, which limits the tunability of the FOMST^6^. In contrast, Fe-doping on Mn will expedite the AFM-FM process and have a wider FOMST temperature range. As a result, the pronounced AFM-FM competition in martensitic state can be realized in the Mn_1-*x*_Fe_*x*_NiGe with fewer Fe amount. For instance, an apparent transition in the *M(T*) curve can be encountered in the samples with *x* = 0.06 and 0.11 for substituting Fe for Mn, while only an inconspicuous transition takes place in the samples with *x* = 0.15 and 0.18 by substituting Fe for Ni^12^.

In Mn_1-*x*_Fe_*x*_NiGe (*x* = 0, 0.06 and 0.11), *T*_*cr*_ gradually decreases with increasing magnetic field, as seen in Figs 3a,c,d and 5a. A reasonable explanation for this is that the Zeeman energy (–*MH*) plays a pivotal role in the AFM-FM competition and conversion. An enhanced Zeeman energy, provided by higher applied fields or greater magnetizations of martensite by Fe-substitution, gives rise to energetically more favorable FM martensite phase^6^. As a consequence, a lower *T*_*cr*_ is obtained under a higher magnetic field or for the sample with more Fe-doping.

The first-order magnetoelastic nature of the AFM-FM-like transition has been proven by the thermal hysteresis (Fig. 3), the standard Arrott plot (Fig. 6) and the *T*_*cr*_ versus *M*^2^ plot (Fig. 5b). To the best of our knowledge, exchange-derived giant magnetoelastic interactions might be general in the systems that possess competing exchange interactions relieved by temperature or applied field^29^. For the Mn_1*-x*_Fe_*x*_NiGe system with low Fe amounts, we speculate that the weak FM ordering competing intensively with the intrinsic AFM coupling may arouse a large magnetoelastic interplay, hence converting a simple AFM-FM-like magnetic transition into a first-order one, as proposed by Wolf^48^. Furthermore, the application of magnetic field will increase the alignment of the magnetic domains in the direction of the external field, which will promote magnetoelastic interaction, leading to a decrease of *T*_*cr*_^49^.

At the end of the last section, the origin of this AFM-FM-like magnetoelastic transition in the low temperature regime was made clear. An Invar-like behavior was observed in the martensitic state of the Fe doped samples, as seen in Fig. 8a. This Invar-like effect may be brought about by the magnetic instability for established FM ordering and a giant change in the two shortest Mn-Mn distances as in the CoMnSi system^29^. Through closer inspection on its enlarged view for the sample with *x* = 0.06 (Fig. 8b), it is found that the sample volume starts to shrink and shortens the Mn-Mn spacing at ~231 K, thus strengthening the AFM exchange during cooling process^21^. As a result, the FM-AFM-like transformation occurs. When the sample is further cooled to ~175 K, the volume begins to expand, consequently enlarges the Mn-Mn spacing, which may again leads to an enhanced FM ordering, as seen in the *M(T*) curve at *H* = 0.5 kOe in Fig. 3b. The fact that volume change is accompanied by the magnetic transition supports the presence of magnetoelastic interaction. Similar magnetic behavior driven by the change of Mn-Mn distance was also reported in Cu-doped MnCoGe recently^50^.

The calorimetric performance of the MT was evaluated by the DSC (Fig. 2) and Δ*S*_*T*_ curves (Fig. 7). For *x* = 0.06, large Δ*S*_*tr*_ but small Δ*S*_*T*_ values are obtained above room temperature. For *x* = 0.11, its larger magnetization variation, together with a much sharper transition, guarantees a larger Δ*S*_*T*_. On the other hand, for the NTE corresponding to the magneto-volume effect of MT, impressive values of ~6500 × 10^−6^ and 4600 × 10^−6^ were achieved during cooling, although it is seriously weakened by the abundant epoxy resin. The optimization of the bonding technique is still underway.

To summarize, double first-order transitions in Mn_1-*x*_Fe_*x*_NiGe (*x* = 0.06, 0.11) alloys were carefully characterized with the aid of the XRD, DSC, magnetization and the linear thermal expansion measurements. The presence of the magnetoelastic interaction during the AFM-FM-like transition in the martensite state was proven explicitly by the linear *T*_cr_(*M*^2^) relation and the tendency of the low-temperature Δ*L*/*L* of the sample with *x* = 0.06. On the other hand, the MCE and NTE performances were evaluated for all these samples and exhibited outstanding potentials. A giant NTE coefficient of −60.7 × 10^−6^/K over *T* = 231–338 K and a near zero thermal expansion of 0.6 × 10^−6^/K over *T* = 175–231 K for the sample with *x* = 0.06 are achieved. These remarkable characteristics make it a good candidate as both NTE and zero-thermal-expansion materials over respective temperature ranges. These findings provide incremental contributions to the understanding of the physical properties of hexagonal *MM*’*X* alloys.

## Methods

Polycrystalline ingots of Mn_1-*x*_Fe_*x*_NiGe (*x* = 0,0.06, 0.11) were prepared by arc-melting under an argon atmosphere using high-purity raw materials. The ingots were annealed at 1123 K in an evacuated quartz tube for five days for homogenization and cooled slowly to room temperature. The as-prepared samples *x* = 0 and 0.06 naturally cracked into small pieces after annealing. Whereas, the sample *x* = 0.11 maintained its initial shape of a button. All samples were ground into powders for the following measurements. The crystalline structure was characterized by room-temperature/high temperature XRD measurements using a Rigaku Ultima IV diffractometer with Cu *K*_*a*_ radiation. The XRD data were analyzed using the Rietveld refinement program FullProf^51^. The phase transitions of the samples were checked by a DSC (TA, Q2000) at a rate of 3 K/min and a vibrating sample magnetometer (VSM, Quantum Design, Versalab-3T) at a rate of 1.5 K/min. The thermal expansion was measured using a standard strain gauge with a resolution of 1 × 10^−6^ at a rate of 3 K/min. In order to fulfill the standard strain-gauge measurements, the sample powders were first mixed with 20 wt.% of epoxy resin as well as hardening agent, and moulded under pressure before subsequent solidification at 443 K.

## Additional Information

**How to cite this article:** Xu, K. *et al*. Magnetocaloric effect and negative thermal expansion in hexagonal Fe doped MnNiGe compounds with a magnetoelastic AFM-FM-like transition. *Sci. Rep.* **7**, 41675; doi: 10.1038/srep41675 (2017).

**Publisher's note:** Springer Nature remains neutral with regard to jurisdictional claims in published maps and institutional affiliations.
